# Efficacy of Antibiotic Therapy Alone Versus Antibiotics With Percutaneous Drainage in Periappendiceal Abscess: A Systematic Review and Meta-Analysis

**DOI:** 10.7759/cureus.73979

**Published:** 2024-11-19

**Authors:** Omeralfarouk H Mohammed, Abubakr Ali M Humidan, Almegdad S Ahmed, Sharwany S Ahmed, Rami F Ibrahim, Malaz Abusefian Elbagir Omer, Muaz Hassan, Mosab Hussen Mostafa Adam, Abdallah M Ali, Shakir Mohamed, Omer H Salim

**Affiliations:** 1 General Surgery, Faculty of Medicine, University of Khartoum, Khartoum, SDN; 2 Microbiology and Parasitology, Faculty of Medicine, University of Khartoum, Khartoum, SDN; 3 General Surgery, Faculty of Medicine, Karary University, Khartoum, SDN; 4 General Surgery, Ribat University Hospital, Khartoum, SDN; 5 General Surgery, Alnao Teaching Hospital, Khartoum, SDN; 6 Orthopedics, University of Khartoum, Khartoum, SDN; 7 Medicine and Surgery, Faculty of Medicine, University of Khartoum, Khartoum, SDN; 8 Soba Teaching Hospital, Faculty of Medicine, University of Khartoum, Khartoum, SDN

**Keywords:** antibiotic therapy, meta-analysis, percutaneous drainage, periappendiceal abscess, recurrence rate

## Abstract

Acute appendicitis is one of the most common surgical emergencies. Simple appendicitis can be a complicated periappendiceal abscess. Despite the fact that there are several treatment options for periappendiceal abscesses, there is no consensus on the optimal treatment method; some surgeons prefer appendectomy, while others prefer relying on non-operative approaches using antibiotic therapy with or without percutaneous drainage. The aim of this study was to compare the efficacy of antibiotics-only therapy to antibiotics plus percutaneous drainage in the management of periappendicular abscess. An electronic database and clinical trial register search was performed on the PubMed, EMBASE, SCOPUS, ClinicalTrials.gov, and Cochrane libraries. After the removal of duplicate records, the studies went through a screening process to determine their eligibility. A meta-analysis was performed for the length of hospital stay and treatment success rate for the comparison groups (antibiotics-only and antibiotics plus percutaneous drainage), in which the mean difference with a 95% confidence interval, and odds ratio using the Mantel-Haenszel method were calculated. The heterogeneity among the studies was assessed using the I^2^ value. Four studies were included in the review and the meta-analysis. Most of the included studies had a retrospective design with the exception of one study, which was a randomized controlled trial. A total of 1,422 patients were included in the four studies; the majority of them (1192, 83.8%) received antibiotics only for the treatment of periappendiceal abscesses, while the rest (230, 16.2%) had percutaneous drainage plus antibiotic therapy. Patients in the antibiotics-only group had a statistically significant mean difference of 2.77 (confidence interval (CI): 3.99-1.55) days of hospital stay less than the percutaneous drainage plus antibiotics group, with a P-value of <0.001. Moreover, they had an average odds ratio of 0.51 (CI: 0.08-3.27) of having more treatment success than the percutaneous drainage plus antibiotics group. However, this was not statistically significant, with a P-value of 0.47. In conclusion, antibiotics-only therapy had a slightly higher odds ratio in terms of treatment success, but this was not statistically significant. In addition, patients in the antibiotics-only group had a decreased length of hospital stay. On the other hand, patients in the antibiotics plus percutaneous drainage group had lower rates of recurrence and required fewer interval appendectomies. More well-designed randomized controlled trials are needed to further solidify the evidence.

## Introduction and background

Acute appendicitis is one of the most common surgical emergencies [1]. Simple appendicitis is usually treated with laparoscopic appendectomy; however, about 30% of the cases can develop periappendiceal abscesses [2]. Management of complicated appendicitis is often associated with increased length of hospital stay, significant morbidity, and high healthcare costs [3]. Despite the fact that there are several treatment options for periappendiceal abscesses, there is no consensus on the optimal treatment method; some surgeons prefer appendectomy, while others prefer relying on non-operative approaches using antibiotic therapy with or without percutaneous drainage [4].

A systematic review stated that no firm conclusions could be drawn regarding the optimal management of periappendiceal abscesses when comparing operative and non-operative options [5]. Another study suggested that compared to appendectomy, non-operative treatment options were associated with fewer complications, such as surgical site infections [6]. There has been a move toward non-operative management of periappendiceal abscesses with antibiotics in addition to percutaneous drainage and surgical washout, and after subsidization of acute inflammation, the patient might undergo interval appendectomy [7,8]. Antibiotic therapy could be beneficial especially in patients with comorbidities [9].

Although there are several reviews comparing operative and non-operative treatment options for periappendiceal abscesses, to our knowledge, this is the first meta-analysis comparing the efficacy of antibiotics monotherapy to antibiotics plus percutaneous drainage in the management of periappendiceal abscesses.

## Review

Methods

This review was performed adhering to the Preferred Reporting Items for Systematic Reviews and Meta-Analyses (PRISMA) guidelines [10]. An electronic database and clinical trial register search was performed on the PubMed, EMBASE, SCOPUS, ClinicalTrials.gov, and Cochrane libraries. The search was done using the following keywords: "periappendiceal abscess", "management", "treatment", "therapy", "therapeutics", "drainage", "surgical procedures", "antibiotic therapy", "conservative management", "percutaneous drainage", and "drainage procedures". These keywords were linked using Boolean operators and other database-specific filtering tools like MeSh terms and others. These sources were searched up to September 2024.

As for the eligibility criteria, we included records of different study designs with the exception of case reports, case series, editorials, letters, commentaries, and conference proceedings. No restriction was applied in terms of the time of publication; however, we only included records in the English language. Included studies had to include two intervention groups for the treatment of periappendiceal abscesses, namely, the antibiotics-only group and the antibiotics plus percutaneous drainage group. They also had to report on the treatment success rate. No restrictions were applied in terms of population age and treatment success parameters.

After the removal of duplicate records, the studies went through the title and abstract screening process to determine their preliminary eligibility. Full texts of records that were deemed preliminary eligible records were retrieved and they were assessed according to the inclusion criteria mentioned above. In addition, reference lists of relevant articles were also screened. Then, a data extraction spreadsheet was used to extract relevant information from the included studies. The extracted data included study design, country, aim, duration, abscess definition, patients' inclusion criteria, number of patients, gender of patients, mean age of patients, antibiotic regimens, and percutaneous drainage procedures, in addition to outcome measures such as treatment success rate, abscess resolution, clinical symptoms resolution, need for surgery following treatment, recurrence rate, length of hospital stay, and others.

A meta-analysis was performed for the length of hospital stay and treatment success rate. The analysis was performed using RevMan 5.4 software (Cochrane Collaboration, Oxford, UK). The mean difference with a 95% confidence interval was used to compare the two groups. Furthermore, the odds ratio for the treatment success rate was done using a random-effects model and Mantel-Haenszel method to compare the two groups. The heterogeneity among the studies was assessed using the I^2^ value; an I^2^ value of more than 75% reflected high heterogeneity among the studies. A P-value of less than 0.05 was considered statistically significant. For other treatment outcome measures, we followed a qualitative approach in the data synthesis. As for the risk of bias assessment, we used RoB 2 and ROBINS-I tools for randomized controlled trials and retrospective studies, respectively [11,12]. 

Results

Study Selection Process

**Figure 1:**
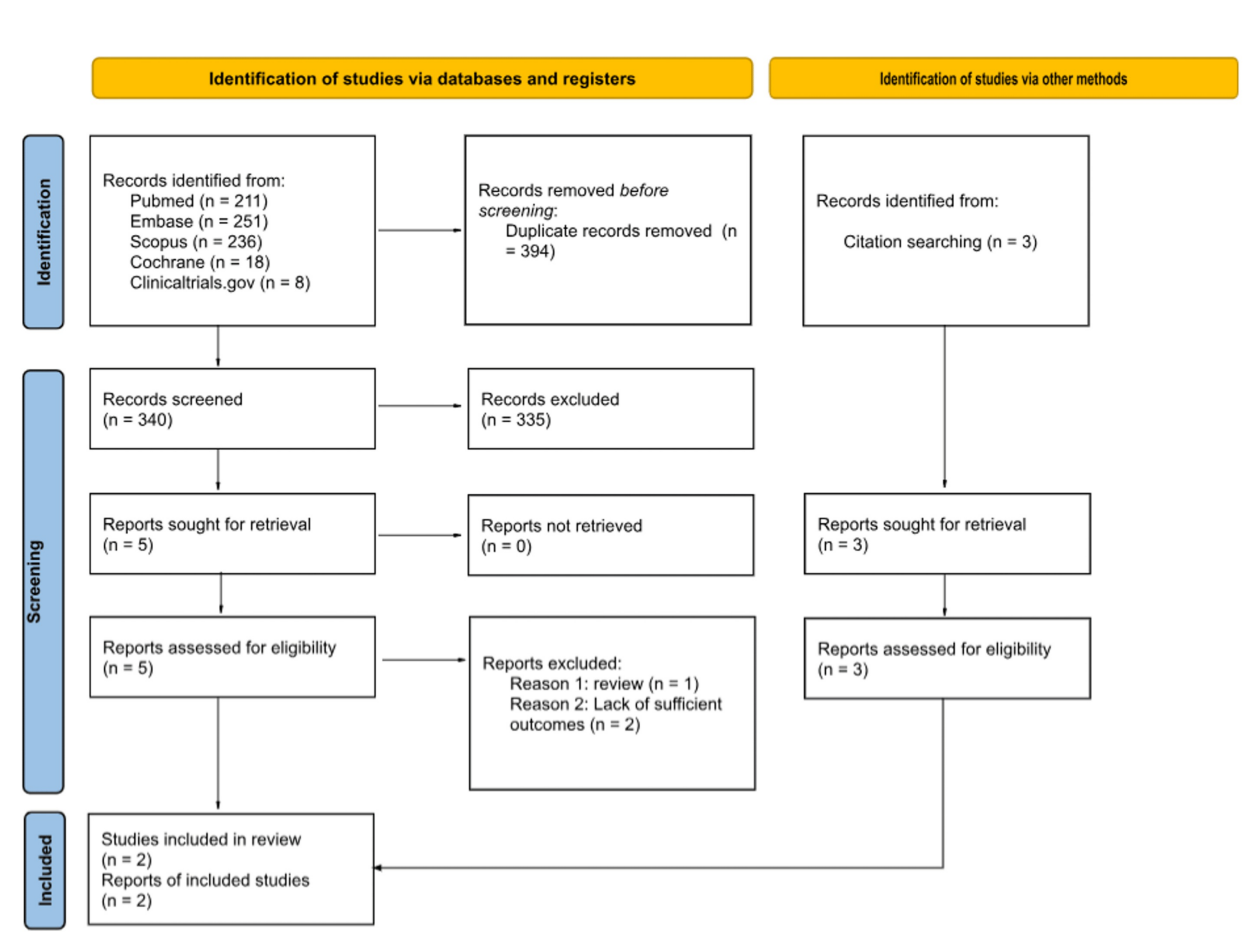
PRISMA flow diagram of the study selection process. PRISMA: Preferred Reporting Items for Systematic Reviews and Meta-Analyse

The electronic databases and registers search identified 734 records. After duplicate removal, these records went through the title and abstract screening process, which resulted in the preliminary inclusion of five studies. After the full-text screening of the five studies, only two of them were deemed suitable to be included in this study. Later on, additional two studies were included in the review after screening the reference lists of relevant records; thus, four studies were included in the review and the meta-analysis. Figure 1 demonstrates the study selection process for this review. 

Basic Characteristics of the Studies and Patients 

Most of the included studies had a retrospective design with the exception of one study, which was a randomized controlled trial [13]. The basic characteristics of the included studies are shown in Table 1. A total of 1,422 patients were included in the four studies; the majority of them (1,192, 83.8%) received antibiotics only for the treatment of periappendiceal abscesses, while the rest (230, 16.2%) had percutaneous drainage plus antibiotics therapy. Moreover, 786 (55.3%) of the patients were males, while 636 (44.7%) were females. The mean age for the patients ranged between eight and 53 years across the included studies. Table 2 shows the patients’ demographic characteristics in the included studies.

**Table 1:** Basic characteristics of the included studies. RCT: randomized controlled trial, CT: computed tomography, HU: Hounsfield units

Study	Country	Design	Aim	Duration	Patient/abscess definition
Zerem (2007) [13]	Bosnia and Herzegovina	RCT	Assessing the effectiveness of percutaneous drainage with antibiotics versus antibiotics alone in treating acute perforated appendicitis	5 years	Patients with acute perforated appendicitis confirmed by symptoms and signs, as well as imaging (ultrasound and/or CT) confirming a periappendiceal abscess. The abscess had to be at least 3 cm in largest diameter. Abscesses larger than 7 cm were confirmed only by ultrasound.
Luo (2016) [14]	Taiwan	Retrospective study	Analyzing the effectiveness of percutaneous drainage therapy in paediatric patients	5 years and 11 months	N/A
Zhang (2019) [15]	United States of America	Retrospective study	Comparing the most commonly adopted nonoperative treatment options (antibiotics with or without drainage)	10 years	Pediatric patients (less than the age of 18 years) diagnosed with complicated appendicitis with abscess formation, confirmed by contrast-enhanced CT demonstrating fluid collection in the right lower quadrant
Chaiyasoot (2021) [16]	Thailand	Retrospective study	Comparing the outcomes of patients with periappendiceal abscess or phlegmon who were treated by different approaches.	6 years	Patients diagnosed with periappendiceal abscess based on CT findings, confirmed by the patients' discharge summary. Periappendiceal abscess is defined as a fluid collection adjacent to the appendix, which has an attenuation of 0-20 HU on CT scan. A phlegmon is defined as an area measuring 20 HU or greater within the periappendiceal fat.

**Table 2:** Characteristics of the included patients. M: male, F: female, N.A.: not applicable, N.S.: not specified

Study	Number of patients	Gender	Mean age (years)	Follow-up duration (month)	Outcome measures
Zerem (2007) [13]	Total: 50 Antibiotics only: 25; antibiotics + drainage: 25	Total: [M:23/F:27] Antibiotics only: [M:12/F:13]; Antibiotics + Drainage: [M:11/F:14]	28.6	24	Resolution of abscess, length of hospital stay, procedure complications, treatment failure, recurrence rate, rate of appendectomy
Luo (2016) [14]	Total: 1,225 Antibiotics only: 1,075; antibiotics + drainage: 150	Total: [M:687/F:538] Antibiotics only: [M:478/F:13]; Antibiotics + Drainage: [M:90/F:60]	N.A	N.A	Appendicitis recurrence rate, need for interval appendectomy, postoperative complications
Zhang (2019) [15]	Total: 46 Antibiotics only: 35; antibiotics + drainage: 11	Total: [M:23/F:23] Antibiotics only: [M:18/F:17]; Antibiotics + Drainage: [M:5/F:6]	8	1-2	Length of hospital stay, complications, treatment response
Chaiyasoot (2021) [16]	Total: 101 Antibiotics only: 57; antibiotics + drainage: 44	Total: [M:53/F:48] Antibiotics only: [M:31/F:26]; Antibiotics + Drainage: [M:22/F:22]	53	N.S. (until complete recovery)	Success/failure of treatment modality, length of hospital stay, readmission rates

Antibiotics-Only Regimens in the Included Studies

All patients in both groups (antibiotics-only group or antibiotics plus drainage) were initially treated with antibiotics. Various types of intravenous antibiotics were used in the included studies including ampicillin, cefuroxime, metronidazole, piperacillin/tazobactam, and ceftriaxone. One study mentioned the use of different antibiotic regimens in the case of intolerance by the patients, namely, ertapenem or meropenem with metronidazole and ciprofloxacin or clindamycin with gentamicin [15]. The duration of the initial intravenous antibiotic-only therapy ranged from five to 14 days. In one study, in the drainage group, the antibiotic regimen was modified according to the culture and sensitivity tests performed on pus aspirated during the drainage procedure [13].

Percutaneous Drainage Procedures in the Included Studies

As for the guidance technique, one of the included studies exclusively relied on using ultrasound guidance [13]. On the other hand, in Zhang et al.'s study, CT-guided drainage was the method of choice [15]. In the other two studies, both methods were used for the drainage. 8F general-purpose catheters were the most used catheters in the included studies, but in Chaiyasoot et al.’s study, 8-12F catheters were used [16]. Two studies employed a transabdominal access route for abscess drainage, while the rest of them did not mention the most commonly used route [15,16]. 

Length of Hospital Stay

**Figure 2:**
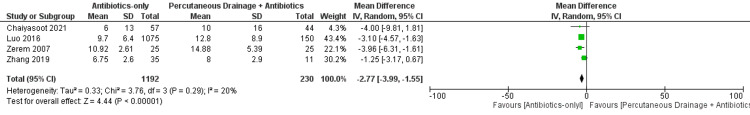
Meta-analysis results for the length of hospital stay comparison for the two groups. References: [13-16]

All of the included studies have investigated the impact of using the two interventions on the length of hospital stay for the patients. Pooled analysis of the data using a random effects model favored the antibiotics-only group, with patients in this group having a statistically significant mean difference of 2.77 (CI: 3.99-1.55) days of hospital stay less than the percutaneous drainage plus antibiotics group, with a P-value of <0.001. The largest and smallest mean difference was reported in Chaiyasoot et al. and Zhang et al. studies, with 4.00 and 1.25 days, respectively [15,16]. Figure 2 shows the meta-analysis results for the length of hospital stay comparison for the two groups.

Treatment Success

**Figure 3:**
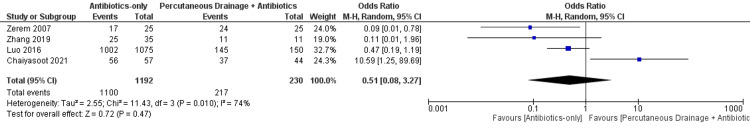
Meta-analysis results for the treatment success comparison for the two groups. References: [13-16]

All of the included studies have investigated treatment success rates for both interventions using various parameters. Pooled analysis of the data using the random effects model also favored the antibiotics-only group, with patients in this group having an average odds ratio of 0.51 (CI: 0.08-3.27) of having more treatment success than the percutaneous drainage plus antibiotics group. However, this was not statistically significant, with a P-value of 0.47. Figure 3 shows the meta-analysis results for the treatment success comparison for the two groups.

Recurrence Rate

Recurrence rate was assessed in three of the included studies [13,14,16]. In Zerem et al.’s study, it was measured at 24% in the antibiotics plus drainage group compared to 48% in the antibiotics-only group [13]. Similarly, the recurrence rate was reported as 3.5% in the antibiotics plus drainage group compared to 7.3% in the antibiotics-only group by Luo et al. [14]. Chaiyasoot et al.'s study also reported similar results, with a recurrence rate percentage of 2.3% in the antibiotics plus drainage group compared to 5.3% in the antibiotics-only group [16]. Overall, the recurrence rate was high with antibiotics monotherapy compared to antibiotics plus drainage.

Rate of Appendectomy

The rate of appendectomy after treatment with both methods was clearly reported in two studies. In Zerem et al.’s study, 36% of the patients in the antibiotics plus drainage group required appendectomy compared to 92% of the patients in the antibiotics-only group [13]. Moreover, the percentage was 7.9% in the antibiotics plus drainage group compared to 19.3% in the antibiotics-only group in Luo et al.’s study [14]. It was also reported in this study that the complication rate following appendectomy was higher in the antibiotics plus drainage group compared to the antibiotics-only group, i.e., 6.1% compared to 0%. 

Risk-of-Bias Assessment and Heterogeneity in the Included Studies

Only one study utilized a randomized controlled study design, so the RoB 2 tool was used in the risk-of-bias assessment [13]. Overall, the study showed a low risk of bias in all domains of the assessment tool. As for the rest of the studies, the ROBINS-I tool was used for the risk of bias assessment. Similarly, all of them had an overall low risk of bias across all domains. As for the heterogeneity, in the pooled analysis of the length of hospital stay, the I^2^ value was 20%, indicating low heterogeneity among the studies (Figure 2). On the other hand, the pooled analysis for the treatment success rate showed relatively high heterogeneity among the studies with an I^2^ value of 74% (Figure 3).

Discussion

Comparing the efficacy of antibiotics-only therapy to antibiotics plus percutaneous drainage, we found that antibiotics had more odds ratio of treatment success, but this was not statistically significant. A meta-analysis that compared surgical and non-surgical treatment options found that antibiotic monotherapy was effective in the management of periappendiceal abscess with a successful outcome in 93% of the patients, with only 20% of the patients [17]. Despite the high success rate, patients in the antibiotics-only group had high rates of recurrence compared to the other group. Many factors have been identified as predictors for recurrent abscess after non-operative management with antibiotics monotherapy including longer duration of intravenous antibiotics, high white blood cell (WBC) count at the time of discharge of more than 8 × 109/L, discharging patients without continuing oral antibiotics, and the ratio of mass size to body surface area larger than 4.3 [18]. 

Regarding the need for appendectomy after treatment, more patients in the antibiotics-only group required interval appendectomies compared to the percutaneous drainage group. The complication rate was also higher among patients who required surgery in the antibiotics-only group. There are several factors that could indicate the need for interval appendectomy, such as increased levels of C reactive protein, the presence of appendicolith, leukocytosis at admission, and small bowel obstruction at the time of admission [19-21]. On the contrary, another study found that leukocytosis at the time of admission was not a reliable risk factor for percutaneous drainage patients [22]. It is worth mentioning that appendiceal perforation, mesoappendix clipping, and elevated CRP levels on admission were associated with an increased risk of abscess development [23,24].

Three of the included studies utilized US guidance, which is considered to be the best guidance modality for percutaneous drainage [25]. Contrast-enhanced ultrasound can be used to provide additional information during the procedure, such as visualizing the microcirculation within the lesions [26,27]. A novel method, i.e., employing dual-pathway contrast-enhanced ultrasound through both blood vessels and drainage tubes in percutaneous drainage, has been developed. This method can assist in better visualization of necrosis and liquefaction of the abscess cavity and provide guidance for placement of the drainage tube [28]. Two of the included studies utilized a transabdominal access route for abscess drainage, and the transgluteal approach was rarely used. It is worth mentioning that the transgluteal approach may increase intraprocedural and postprocedural pain, which might affect patient satisfaction [29,30]. 

One of the limitations of this study was the few number of the included studies, and the majority of them had a retrospective design, which carries an increased risk of bias, including selection bias and recall bias. Thus, more well-designed randomized controlled trials are needed to solidify the evidence. Another limitation was the high heterogeneity among the studies evidenced by the high I^2^ value, especially in the pooled analysis for the treatment success comparison. This could be due to the difference in populations of the included studies. It could also be due to differences in the parameters used to measure the treatment success rate and different antibiotic regimens used in the studies. Some studies relied on abscess resolution using clinical and imaging indicators, while others measured recurrence rate as an indicator of treatment success.

## Conclusions

The aim of this study was to compare the efficacy of antibiotics-only therapy to antibiotics plus percutaneous drainage in the management of periappendicular abscesses. The antibiotics-only therapy had a slightly higher odds ratio in terms of treatment success, but this was not statistically significant. In addition, patients in the antibiotics-only group had a shorter length of hospital stay. On the other hand, patients in the antibiotics plus percutaneous drainage group had lower rates of recurrence and required fewer interval appendectomies. However, more well-designed randomized controlled trials are needed to further solidify the evidence.
